# Functional states shape the spatiotemporal representation of local and cortex-wide neural activity in mouse sensory cortex

**DOI:** 10.1152/jn.00424.2021

**Published:** 2022-08-17

**Authors:** Miriam Schwalm, Dennis R. Tabuena, Curtis Easton, Thomas J. Richner, Pierre Mourad, Hirofumi Watari, William J. Moody, Albrecht Stroh

**Affiliations:** ^1^Institute of Pathophysiology, University Medical Center Mainz, Mainz, Germany; ^2^Department of Biological Engineering, Massachusetts Institute of Technology, Cambridge, Massachusetts; ^3^Department of Biology, University of Washington, Seattle, Washington; ^4^Leibniz Institute for Resilience Research, Mainz, Germany

**Keywords:** brain states, calcium imaging, LFP, mouse, sensory cortex

## Abstract

The spatiotemporal representation of neural activity during rest and upon sensory stimulation in cortical areas is highly dynamic and may be predominantly governed by cortical state. On the mesoscale level, intrinsic neuronal activity ranges from a persistent state, generally associated with a sustained depolarization of neurons, to a bimodal, slow wave-like state with bursts of neuronal activation alternating with silent periods. These different activity states are prevalent under certain types of sedatives or are associated with specific behavioral or vigilance conditions. Neurophysiological experiments assessing circuit activity usually assume a constant underlying state, yet reports of variability of neuronal responses under seemingly constant conditions are common in the field. Even when a certain type of neural activity or cortical state can be stably maintained over time, the associated response properties are highly relevant for explaining experimental outcomes. Here we describe the spatiotemporal characteristics of ongoing activity and sensory-evoked responses under two predominant functional states in the sensory cortices of mice: persistent activity (PA) and slow wave activity (SWA). Using electrophysiological recordings and local and wide-field calcium recordings, we examine whether spontaneous and sensory-evoked neuronal activity propagate throughout the cortex in a state-dependent manner. We find that PA and SWA differ in their spatiotemporal characteristics, which determine the cortical network’s response to a sensory stimulus. During PA state, sensory stimulation elicits gamma-based short-latency responses that precisely follow each stimulation pulse and are prone to adaptation upon higher stimulation frequencies. Sensory responses during SWA are more variable, dependent on refractory periods following spontaneous slow waves. Although spontaneous slow waves propagated in anterior-posterior direction in a majority of observations, the direction of propagation of stimulus-elicited wave depends on the sensory modality. These findings suggest that cortical state explains variance and should be considered when investigating multiscale correlates of functional neurocircuit activity.

**NEW & NOTEWORTHY** Here we dissect the cortical representation of brain states based on local photometry recordings and on mesoscale cortical calcium imaging, complemented by electrophysiological recordings in mice. We identify two distinct functional states in the sensory cortices, which differ in their spatiotemporal characteristics on the local and global cortical scales. We examine how intrinsic and stimulus-evoked neuronal activity propagates throughout the cortex in a state-dependent manner, supporting the notion that cortical state is a relevant variable to consider for a wide range of neurophysiological experiments.

## Graphical abstract

**Figure:**
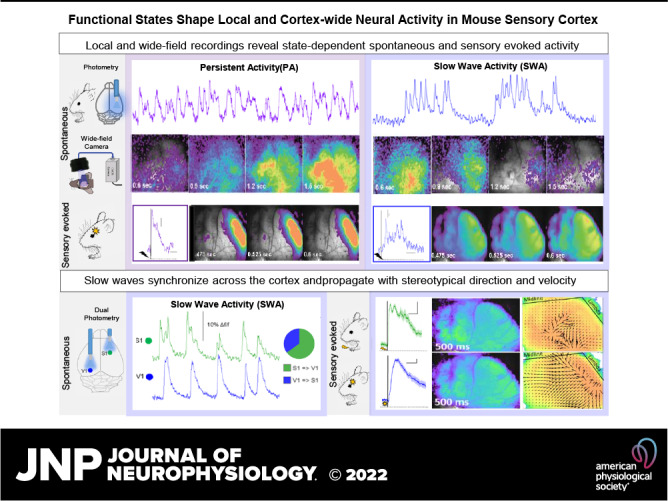

## INTRODUCTION

Functional neural networks undergo constant fluctuations elicited by changes of internally generated activity, even in the absence of external stimulation (1, 2). Alternating states of excitability are associated with changes in global functional condition, as between sleep and wakefulness (3, 4), from inattention to vigilance (5, 6), or from resting to locomotion (7, 8). These changes impact the spatiotemporal organization of ongoing neuronal signals and shape responses upon incoming sensory information on multiple spatial scales (8–10). State-dependent shaping of intrinsic and stimulus-evoked signals occurs on the level of single neurons (11–13) or local circuits (14) and can influence global functional connectivity (15). The spatiotemporal representation of neural activity in cortical areas therefore must be state dependent and dynamic. Active cortical states are generally associated with a sustained depolarization of cortical neurons (3, 14, 16) and suppression of silent states (4, 6, 17), leading to fast, ongoing fluctuations of neural activity. Such persistent activity is often present during awake periods (2, 18, 19), but a similar state may also be maintained by certain sedatives (14, 15, 20). Conversely, bursts of neural activity alternating with silent periods can be found under a variety of different conditions in populations of thalamocortical neurons (7, 21–26). These bimodal patterns of activity originate in deeper layers of the cortex (27–29) and were shown to affect neuronal excitability as well as evoked perturbations of neuronal networks throughout the brain (6, 12, 13, 17, 24, 30, 31). Such slow wave-like activity can occur rather locally (32) and can spread over the entire cortex in rodents (15, 20, 29) or over large areas in primates (33), while being spontaneously initiated mostly in frontal areas (29, 34, 35). Slow waves have classically been described during deep anesthesia (23, 28–30, 36–39), but a comparable type of activity may also be found during awake resting (17, 40, 41). Although their main features are similar to those of slow-wave sleep (SWS) waves (3, 23), slow waves under anesthesia occur more rhythmically and synchronously across the cortex, show longer periods of inactivity between waves than during SWS (36, 37), and represent a biologically and potentially clinically significant neural phenomenon (33). Persistent activity (PA) and slow wave activity (SWA) are both used as underlying conditions in neurophysiological studies that are performed under anesthesia or sedation (42), and variations of these states can similarly occur in awake animals. The current underlying cortical state might represent an important variable both for anesthetized preparations and for experiments performed in awake animals (17). Previous work has explored the spatiotemporal dynamics of cortical sensorimotor integration by relating motor and barrel cortex activity in both freely moving (9) and head-fixed (10) mice by voltage-sensitive dye imaging. Neuronal activity in mouse visual cortex was shown to differ between quiescence and locomotion (43–48) but can also differ without a change of behavioral state, likely depending on spontaneous changes of cortical network activation (6, 17, 49–51). For auditory responses, similar influences of behavior (52) as well as of anesthesia-induced brain state (53) have been demonstrated. In a recent study, local electrophysiological activity was compared for different anesthesia paradigms (42). Here, we use optical and electrophysiological signals, locally recorded in the primary visual and somatosensory area, and wide-field camera imaging of the entire mouse cortex to compare and assess two distinct functional states, which are relevant for a variety of neurophysiological studies: a state dominated by SWA, typified by bimodal activity of relative quiescence interrupted by bursts of activity, and a persistently active (PA) state with constant, uninterrupted activity. In our study, these two states are mimicked by deep isoflurane anesthesia and medetomidine sedation, respectively, both frequently used in neuroscience and especially in rodent neuroimaging studies, where anesthetized animals are most commonly the norm (54–56). We characterize the respective neuronal activity related to each state in all modalities and examine how the ongoing signal, as well as sensory-evoked responses, recruit the cortex. We find that SWA and PA differ substantially regarding spatiotemporal characteristics on the local and global scales and show state-dependent propagation modes. As different types of anesthesia as well as different behavioral states can show characteristics similar to the two states described here, SWA and PA represent an experimental model for cortical information processing and are relevant for explaining response variability of neural networks under seemingly constant conditions.

## MATERIALS AND METHODS

### Animals

All animals were housed under a 12:12-h light-dark cycle and provided with food and water ad libitum. Animal husbandry and experimental manipulation were carried out according to international standards and were approved either by the Landesuntersuchungsamt Rheinland-Pfalz, Koblenz, Germany or by the University of Washington (UW) Institutional Animal Care and Use Committee. Acute photometry and/or local field potential (LFP) experiments were performed on 17 adult BL6 mice (>8 wk old), in 7 of which we performed awake recordings on a tracking ball. LFP recordings with implanted electrodes were performed on 10 wild-type (WT) mice. Wide-field camera experiments were performed on nine animals, of which five were crosses of Cux2-Cre-Camk2-tet and Ai93 and four were Emx1-Cre and Ai162. We did not observe epileptiform activity (57, 58) in any of the transgenic animals used for this study. To generate these animals, two different types of transgenic mice were used. The first were triple-transgenic animals expressing cre/tet-dependent GCaMP6f (Ai93, Jackson Laboratories, stock no. 024103) as well as Cux2-Cre and Camk2-tet. The second group of animals expressed cre-dependent GCaMP6s (Ai162, Jackson Laboratories, stock no. 031562) and Emx1-Cre. It should be noted that there were survival issues with the second animals, likely due to the toxicity in response to excessive expression levels. However, a subset of animals grew up to healthy adults, and no differences were observed in the patterns of activity propagation versus the Ai93 animals. The increased calcium fluorescence in the Ai162 animals allowed faster image acquisition with increased signal-to-noise ratio.

### Fiber Photometry

Mice were anesthetized with isoflurane mixed with pure oxygen to maintain surgical depth of anesthesia (3.0% induction, 1.5–2.0% maintenance of anesthesia). Animals were fixed in a stereotactic frame with ear bars and bite bars and placed onto a warming plate to keep body temperature constant at 37°C throughout the surgery. Anesthesia depth was repeatedly assessed by monitoring the tail-pinch reflex as well as the respiration rate of the animal. After application of topical anesthesia (xylocaine gel 2%; AstraZeneca, Wedel, Germany) on the animal’s head, a skin incision was made and the exposed area of the skull was cleaned and dried of fluids and blood. For anesthetized experiments involving recordings in somatosensory (S1) or visual (V1) cortex, lateral muscles were partially removed with the help of a scalpel. Stereotactic coordinates of the regions targeted for dye injection were located accordingly [coordinates for mice were taken from Paxinos and Franklin (59)], and a craniotomy was prepared under a dissecting microscope (Leica, Wetzlar, Germany) with a dental drill (Ultimate XL-F; NSK, Trier, Germany, and VS1/4HP/005; Meisinger, Neuss, Germany). For calcium dye injections a small burr hole was opened. The bone was removed with a sharp scalpel or a needle, the exposed area was moistened with phosphate-buffered saline (PBS; Sigma, Munich, Germany), and the fluorescent calcium indicator Oregon Green BAPTA-1 AM (OGB-1; Life Technologies/Molecular Probes, Waltham, MA) was prepared as previously described (83) and bulk loaded into the somatosensory (S1; AP −0.2 mm, ML +/−2.5 mm, DV −300 and −500 µm) and visual (V1; AP −3.8 mm, ML +/−2.0 mm, DV −300 and −500 µm) cortex. Dye solution was delivered with a glass micropipette with an outer tip diameter of 45 µm and an inner diameter of 15 µm connected to a 10-mL syringe. Approximately 0.5–1.0 µL of the solution was slowly released at each injection depth by gentle manual pressure. After injection, the pipette was held in place for 2 min before being slowly retracted from the tissue. After a waiting period of 50–60 min to allow for ester cleavage, the cladding from the tip of a multimode optic fiber with a 200-µm diameter and 0.48 numerical aperture (Thorlabs, Grünberg, Germany) was removed and the fiber was inserted perpendicular to the dura above each recording site (S1 and ipsi- or contralateral V1). For anesthetized recordings mice were kept in the stereotactic frame, and the fibers were held in place by two stereotactic arms. Mice for awake recordings either underwent a previous surgery 3 days before the experiments for implantation of a metal holder on the cranium or were implanted with such a holder directly during the dye injection and fiber implant surgery. The metal holder was fixed on the skull with dental cement. For awake recordings fibers were glued to the skull with UV glue. For excitation of the calcium indicator, we used custom-built setups coupling a LED directly into an optic fiber (FOM and FOMII; NPI Electronic Instruments, Tamm, Germany). The light for excitation of the calcium indicators was delivered by a 650-mW LED with a nominal peak wavelength of 470 nm. LED power was controlled by an adjustable current source. The light beam was focused by means of a fixed-focus collimator into one end of a multimode fiber, which was connected to the system by an SMA connector. An aspheric lens was used to guide the emitted fluorescent light back through the same fiber, where it was focused on a silicon photomultiplier photodetector with an active area of 3 × 3 mm^2^ and a photon detection efficiency of 20% at 490 nm. The recorded fluorescence signals were digitized with a sampling frequency of 2 kHz using a multifunction I/O data acquisition interface (Power1401; Cambridge Electronic Design, Cambridge, UK) and its corresponding acquisition software (Spike2). For excitation of the calcium indicator, we used constant illumination of 1.3 mW/mm^2^ at a wavelength of 470 or 488 nm, which was switched on at the beginning and stayed on during the course of the measurement. For somatosensory stimulation under anesthesia, two needle electrodes were subcutaneously inserted into the forepaw (between digits 2 and 4), contralateral to the recording site, and connected to a constant-current stimulator (DS5; Digitimer, Welwyn Garden City, UK). Stimulation pulses were of constant duration (10 ms) and strength (1 mA). Either single-pulse stimulation or a block paradigm with a pulse train of 4 s at 3 Hz, followed by 10-s or 20-s baseline, was used. For visual stimulation in anesthetized or awake mice, a TTL pulse-controlled small LED was used on the eye contralateral to the recording site. Depending on the experiment, either single-pulse stimulation (10-ms light pulses) or a block paradigm as described for somatosensory stimulation was used. For awake recordings, after animals recovered from anesthesia on the tracking ball, the airflow controlling the Styrofoam ball was set to let animals run if they chose to initiate movement while they were head fixed with a metal holder that was attached to a frame. Calcium data were preprocessed with IGOR Pro and were low-pass filtered with a 20-Hz Gaussian filter before subsequent event detection if not stated otherwise. All depicted traces represent relative changes in fluorescence intensity (df/f). Events were treated as experimental units given their stereotypical properties, which are ubiquitously observed across animals. The descriptive statistics of the event properties distributions grouped by animals were very similar across individuals. Thus, calcium response statistics were pooled over events (mathematically equivalent to weighted averages), ensuring that every event is contributing equally to the average, independent of its source (the individual animal). Data were tested for normal distribution with the Lilliefors test, an assumption-free adaptation of the one-sample Kolmogorov–Smirnov test. In cases in which normal distribution could be assumed (*P* > 0.05), the parametric two-tailed Student’s *t* test was employed to compare means (a *P* value < 0.05 was considered significant). Where normal distribution could not be verified, the nonparametric Wilcoxon rank-sum test for equal medians was used to test median differences. All quantifications are presented as means ± SE, unless stated otherwise. In cases of an unstable baseline or absence of characteristic signal dynamics of slow wave-associated calcium transients (29, 53) calcium data were excluded from analysis. The time series trace from the fiber data was denoised with binomial (Gaussian) smoothing and normalized by dividing the original trace by the averaged baseline, for which 1 s of baseline was chosen manually. Latencies were quantified semiautomatically in each poststimulus response. Cross-correlations of calcium signals were computed with the function *crosscorr* in MATLAB. To determine the length of the refractory period, the rate of successful slow wave generation in response to a sensory stimulus was calculated with respect to time elapsed from the previous spontaneous wave. This time was measured for each stimulus and binned into 0.5-s intervals. The first interval at which >90% of sensory stimuli resulted in a slow wave in the associated sensory cortex was calculated at 2 s. This calculation pooled somatosensory and visual stimuli across animals. For the comparisons of stimulus responses with shuffled stimulus onsets, photometry signals were linearly detrended and then averaged by the depicted window around the real stimulus onset or the temporally shuffled stimulus onset times. For the latter, stimulus onsets were shuffled randomly within a range of 2 s around each real stimulus onset, for the same number of stimulus events, respectively. Latencies for single-pulse responses during PA were defined as the maximum amplitude within a 2-s window after the real (or temporally jittered) stimulus onset.

### Wide-Field Imaging

For these experiments a titanium U-shaped head-holder was used instead of ear bars. A craniotomy was performed, and the U bar was positioned and glued to the surface of the skull with cyanoacrylate glue such that a majority of one hemisphere was exposed. The U bar was held in place with an adjustable clamp, and a Dremel was used to gently remove the flaky layers of the skull until blood vessels were clearly visible beneath the remaining skull when moistened. Then the skull was allowed to dry briefly, and glue was applied to prepare the surface for imaging. The clamp was adjusted to present a level surface for imaging, and epifluorescent wide-field imaging was used to collect images of GCaMP fluorescence emission at 10–40 Hz, with a Hamamatsu Orca Flash 2.8 CMOS camera and HCImage V4.4.0.11. We used an AD Instruments PowerLab 4/26 and LabChart V8 to trigger and synchronize stimuli to calcium records. To analyze stimulus responses, we first spatially downsampled and calculated the relative change in fluorescence over time (%df/f) for each stimulus, using the frame before stimulus onset as a reference frame. These responses were temporally aligned to stimulus onset and averaged to create stimulus-triggered averages for each animal. To calculate response area, we used a threshold of 3.5 standard deviations of the averaged prestimulus signal to determine the onset of response (if any) on a pixel-by-pixel basis. We used the maximum number of pixels activated within 1–2 s to define the response area. To calculate propagation velocity and direction, we analyzed the difference in latency of neighboring pixels by creating a response time map and then taking the spatial gradient. To compare velocity between visual and somatosensory responses we combined the response vectors of all animals into a weighted polar distribution after manually aligning images to the midline suture. Distributions were compared with a Kuiper test available in the Circular Statistics Toolbox for MATLAB (60). In addition, we also quantified the mean velocity vector for somatosensory and visual responses by averaging all velocity vectors within each triggered average.

### Local Field Potential Recordings

For acute LFP recordings, a pipette with a tip resistance of 0.2 M was filled with PBS and lowered into either the S1 or the V1 craniotomy at a depth of 300 µm. A grounding wire was implanted in the olfactory bulb. Signals were amplified with a gain of 100, with an extracellular amplifier (EXT-02F/2; npi Electronics, Tamm, Germany), filtered at 300 Hz (low pass), and digitized at 2 kHz. Signals were analyzed with Spike2 software or custom-written MATLAB scripts, including the Chronux toolbox (http://chronux.org). Ongoing neuronal activity during different brain states or individual responses upon sensory stimulation were plotted against time to observe signal characteristics. Spectrograms showing time-frequency profiles were plotted with the Chronux package for multitapers using the following parameters: fpass = 0–40 Hz; Time-Bandwidth-product = 4; number of tapers = 9, and a moving window of 400-ms window size and 200-ms step size. For ongoing activity without stimulation the average for at least 60 s of recording was used; for stimulus-locked spectrograms the responses of 30 stimulus trials were averaged.

For acute LFP recordings using intracranial electrode implants, animals were placed in the stereotax as described above, two small craniotomies were opened, and a silver-wire electrode soldered on a eight-pin head mount was placed at stereotactically determined coordinates: −0.2 AP, 2.5 ML (S1) and −3.8 AP, 2.0 ML (V1). A reference wire was placed between the skin and the skull rostral to the recording electrodes. A four-channel analog adapter recorded the data with PowerLab monitored by LabChart (AD Instruments, Sydney, Australia), and the data were not filtered during acquisition. Here, analysis was conducted on stimulus-triggered response averages from multiple animals. A minimum of 10 responses were averaged for each data point, with each data point representing one animal. Fold change in power was calculated by taking the average power of each response average from a time 0–1.5 s after the stimulus and dividing by the average power in a 200-ms interval ending 100 ms before the stimulus. For separation of slow-wave responses into refractory and nonrefractory groups, an analysis of network history at the time of each stimulus was performed. The V1 electrical record was converted to power over time, and slow-wave onsets were detected where the power crossed 4 standard deviations above the mean. A stimulus response was considered to have occurred in the refractory period if spontaneous event onset was detected <2 s before stimulus.

### Anesthesia

To reach the functional state under investigation, different anesthesia protocols were used. Slow wave activity emerges under various anesthetics including ketamine-xylazine, propofol, midazolam, halothane, isoflurane, and urethane (e.g., Refs. 37, 61–63), even though there are differences regarding the length of silent periods depending on the type and depth of the anesthesia used. For investigating slow wave activity, isoflurane was continuously supplied through custom-made anesthesia masks equipped with a tooth holder to reduce head movements. We used relatively high levels of isoflurane concentration to maintain slow wave activity (1.2–1.8%) because we were aiming to achieve slow waves around 0.1 Hz. As depth of anesthesia is highly dependent on the physiological state of the animal, e.g., body and therefore cortical temperature (16, 64), we relied on the online calcium or electrophysiological signal to ensure stable slow wave activity throughout the measurements and adjusted the isoflurane level accordingly, to achieve a rather uniform occurrence of slow waves. Consequently, although the absolute level of isoflurane anesthesia varied, based on many parameters such as length of anesthesia, we achieved rather constant conditions in terms of the characteristics of slow waves while the isoflurane concentrations were comparable with others (29, 53, 65, 66, 82). Frequency of spontaneous waves is strongly dependent on the depth of anesthesia, ranging from ∼10 waves/min at high anesthesia levels to 30 waves/min at low levels (29). Further reduction of anesthesia leads to irregular patterns of activity indicating transition to a persistent state, which is usually reached at 0.4–0.6% isoflurane. For maintaining a stable persistent network activity, we either used such low levels of isoflurane (0.4–0.6%) or the sedative medetomidine (Dormitor, 1 mg/mL, Pfizer; Orion Pharma, Espoo, Finland) as a bolus injection of 0.05 mg/kg. For anesthesia controls we used ketamine-xylazine (ket/xyl; 180 mg/kg and 0.011 mg/kg, respectively, in NaCl solution). Physiological parameters such as breathing rate and temperature were continuously monitored. Functional state was assessed with either of the reported recording techniques, and recording sessions in which functional state was unclear or varying were excluded from the analysis.

## RESULTS

### Mesoscale Assessment of State-Dependent Activity

**Figure 1.**
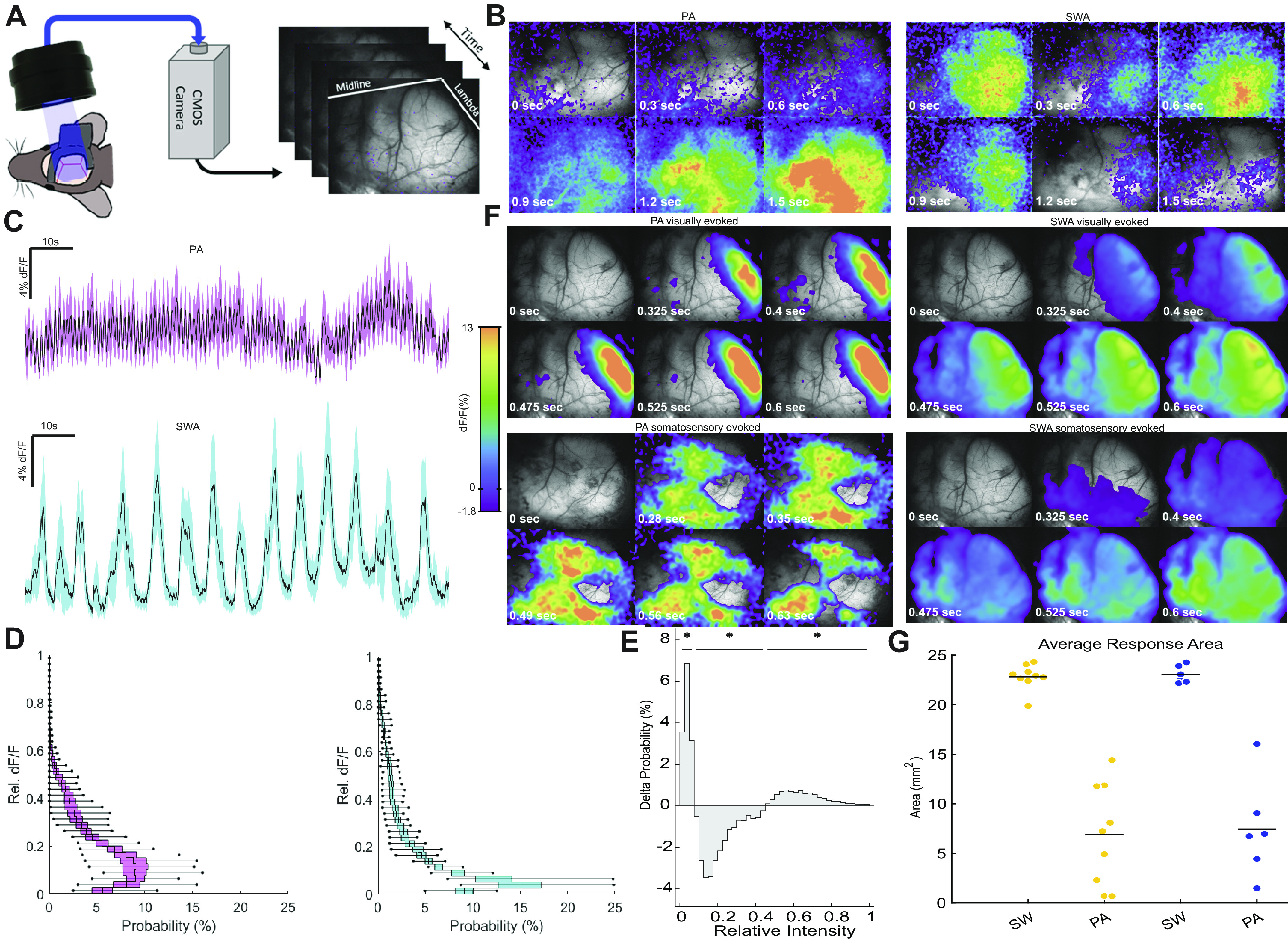
Camera recordings of transgenic GCaMP6 animals reveal mesoscale spatiotemporal characteristics of two distinct activity states. *A*: camera recording setup. *B*: images of different time points during recording of ongoing activity during persistent activity (PA) and slow wave activity (SWA) states. *C*: mean %df/f across cortical field of view for PA state (*top*) and SWA state (*bottom*) shown in *B*. Shaded region indicates the SD at each time point. *D*: average probability distributions of mean df/f across animals. Center lines indicate mean probability for each bin, boxes indicate SD, and whiskers indicate minimum and maximum values (*n* = 9 animals). *E*: difference of the mean probability distributions (shown in *D*): positive probabilities indicate higher occurrence during SWA state; negative probabilities indicate higher occurrence in PA state. Significant regions were determined by permutation-based testing and are indicated by horizontal bars with asterisks (*P* < 0.0001 for all after Bonferroni correction). *F*: propagation properties for sensory-evoked activity in both states: a brief 10-ms LED light flash to the left eye for visual and a mild electric stimulus (1 mA, 10-ms pulse) to the right forepaw for somatosensory; between 13 and 88 stimuli (40 ± 0.7) for each stimulus modality. *G*: average cortical area activated upon visual and somatosensory stimuli in SWA and PA states.

We subjected two different strains of transgenic GCaMP6 mice (see materials and methods) to two sedative regimens, which were previously shown to yield distinct local and brain-wide activity patterns (15, 20). We explored mesoscale correlates of the two activity patterns (SWA and PA) with thinned-skull preparations and wide-field CCD camera imaging techniques (Fig. 1*A*). High-spatial resolution images of almost the entire cortical surface allowed us to observe the spatial activation pattern in each state. We found activity signatures sharing key characteristics of the two activity states previously described by functional connectivity analyses (15): ongoing activity during PA displayed rapid fluctuations with spatially diverse activity over the course of the recording. Short bursts of fluorescence changes were observed across the field of view that were not synchronized in space or time (Fig. 1*B*, Supplemental Video S1). In contrast, the ongoing signal during SWA was characterized by slow-propagating waves of fluorescence. In Supplemental Video S2 (still frames in Fig. 1*B*), a single propagating burst of activity spanning the entire field of view can be observed. Spatial averaging of the field of view (FOV) revealed distinct signal dynamics (Fig. 1, *C*–*E*). We assessed the signal change for each pixel in the FOV to examine the spatial and temporal differences between PA and SWA (Fig. 1*C*). For SWA, we observed large-amplitude oscillations and near-synchronous activation of the entire FOV. For PA, fast and small-amplitude fluctuations of fluorescence were dominant, and the FOV was never fully active or fully quiescent. To quantify these observations, we calculated the probability distribution of the mean fluorescence signal trace with respect to time (Fig. 1, *D* and *E*). The probability distribution of SWA had the largest mass at near-zero activation with a shift to higher activation. PA showed a distribution with two maxima, one at ∼10% and one at 40% activation. The differences in these distributions were significant when compared with permutation-based tests. Next, we explored responses upon sensory stimulation under PA and SWA conditions. We subjected the animals to visual stimulation, light flashes administered to the contralateral eye at an interevent interval of 2–4 s, and somatosensory stimulation, electric stimulation of the hind paw with an interevent interval of 2–4 s. Stimulation current was manually titrated for each animal to induce a cortical response but not to cause a reflexive twitch (1 mA on average). Wide-field time-lapse imaging of sensory responses during PA revealed a spatially confined response to both sensory stimuli (Fig. 1*F*). Upon visual stimulation, an area of activation spanning 6.9 ± 1.7 mm^2^ (*n* = 9 mice) was observed. Upon somatosensory stimulation, an area of 7.5 ± 2.0 mm^2^ (*n* = 6 mice) was active. During SWA, the area of somatosensory-evoked activation spanned 23.1 ± 0.3 mm^2^ (*n* = 6 mice) and 22.8 ± 0.1 mm^2^ (*n* = 6 mice) for visually evoked activity (Fig. 1*G*). These differences in extension of fluorescence change were significant for both conditions (*P* < 0.001, Student’s *t* test for visually and somatosensory evoked fluorescence changes in PA vs. SWA). There was no statistically significant difference between the response areas for visual and somatosensory stimuli within SWA (*P* = 0.687) or PA (*P* = 0.827) state.

### Assessment of Local Signal Dynamics in PA and SWA States

**Figure 2.**
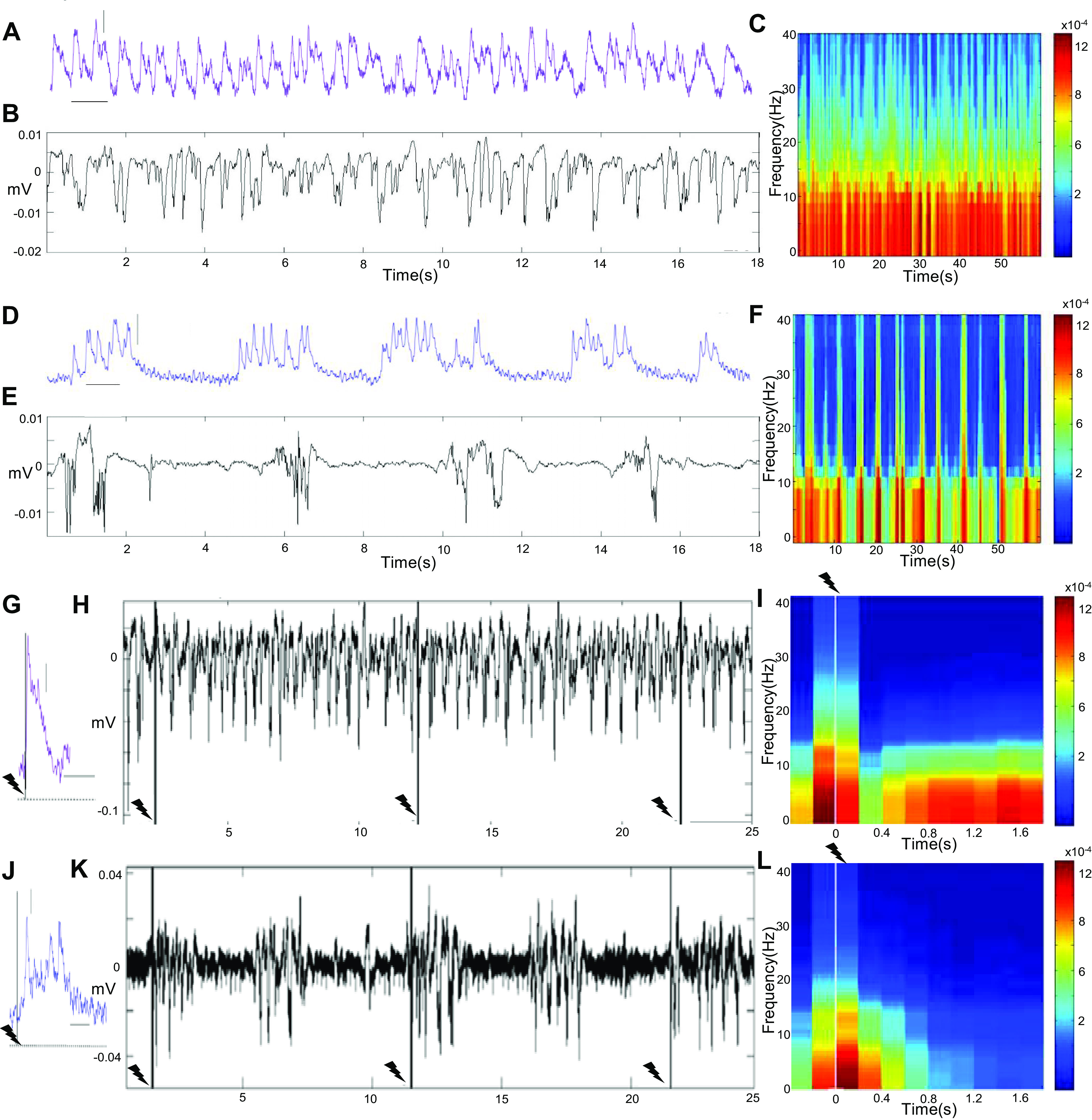
Local dynamics of slow wave activity (SWA) and persistent activity (PA) stated. *A*: photometry recording in somatosensory cortex (S1) during PA state induced by medetomidine sedation (purple trace; acute Oregon Green-BAPTA-1 AM staining). *B*: local field potential (LFP) signals under the same conditions show similar signal dynamics (same timescale as in *A*). *C*: the spectrogram of the LFP signal in *B* reflects the ongoing persistent activity, showing continuous frequencies around 15 Hz. *D*: photometry recording in S1 during isoflurane-induced SWA state (blue trace; acute Oregon Green-BAPTA-1 AM staining) in which stereotypical, long-duration calcium waves interrupted by silence periods become apparent. *E*: LFP signals under the same conditions show similar dynamics (same timescale as in *D*). *F*: the spectrogram of the LFP signal in *E* reflects the alternation of active and silent periods in the frequency distribution, with the appearance of higher frequencies (around 40 Hz) during slow wave events. *G*: electric forepaw stimulation pulses (10 ms, 1 mA) reliably evoke calcium response in PA state (single trial response). Vertical scale bar, 0.02% df/f; horizontal scale bar 0.5 s. *H*: stimulus pulses evoke short-latency LFP spikes during PA state (note that these deflections occur very briefly after the stimulus pulse and for a very short duration in the depiction chosen to be comparable to *K*). *I*: the stimulus-locked spectrogram of the LFP trace in *H* shows time-frequency profiles before and after stimulation pulse (*n* = 30 events). *J*: during SWA state the same stimulus pulse evokes a slow calcium wave with a mean probability of 90% (*n* = 4 animals) that differs in latency, duration, and microarchitecture from an evoked response during PA state in the same animal as shown in *G*; scale bars same as in *G*. *K*: the response properties observed for the calcium signal in *J* also account for the LFP signal during SWA state. *L*: the stimulus-locked spectrogram of the LFP recording in *K* shows time-frequency profiles before and after the stimulation pulse (*n* = 30 events).

To characterize temporal characteristics of ongoing and sensory-evoked activity during SWA and PA with high temporal resolution, we employed single optic fiber-based calcium and local field potential (LFP) recordings in wild-type (WT) mice injected with an optical dye. During PA, continuous signal dynamics in photometry and LFP transients were observed, devoid of prolonged periods of quiescence (Fig. 2, *A* and *B*). The corresponding LFP spectrogram shows continuous power values around 15 Hz (Fig. 2*C*). SWA was characterized by stereotypical calcium waves and LFP deflections, which were interrupted by silent periods (Fig. 2, *D* and *E*), as reported previously by us and others (15, 20, 23, 29, 36, 37, 53, 67). The LFP spectrogram reflects the alternation of slow wave events and silent periods in the frequency distribution, showing high power during slow wave events (Fig. 2*F*). Next, to explore whether the observed differences in the ongoing signals have impact on the neuronal encoding of sensory stimuli, neuronal responses upon forepaw stimulation were compared during PA and SWA states. During PA, short-delay responses become apparent upon electric forepaw stimulation pulses (1 mA, 10 ms; Fig. 2, *G* and *H*), whereas during SWA state forepaw stimuli reliably evoked slow wave events with longer latency and duration (mean probability 90%; *n* = 4 mice; Fig. 2, *J* and *K*). During SWA state forepaw stimulus trains of 3 Hz for 4 s led to calcium waves comparable to those evoked by single pulses, whereas during PA state short-latency responses were elicited by every stimulus pulse upon the same stimulation paradigm (LFP recordings reveal similar signal characteristics; Supplemental Fig. S1). To explore whether functional state dynamics have influences on sensory responses in awake mice, animals with implanted optic fibers for calcium photometry recordings in S1 and V1 were head fixed on a tracking ball, which allowed them to initiate running voluntarily throughout the course of a measurement. Visual stimulation in awake animals led to similar short-latency responses in V1 as previously shown under the light sedative PA state condition (Supplemental Fig. S2, *A* and *B*) and to responses of longer latency when the animal was resting (Supplemental Fig. S2*B*), results well in line with electrophysiological studies (44–46, 48). Importantly, the reported properties of ongoing activity are not limited to the type of anesthesia used for eliciting the two functional states, SWA state is known to be reliably evoked under ketamine-xylazine anesthesia (23, 37, 68) or urethane (63), and PA state can be evoked by low dosages (0.4–0.6%) of isoflurane (15) and shares properties of awake signal characteristics (Supplemental Fig. S2, *C*–*E*).

### The Role of Functional State for Cortico-Cortical Sensory Processing

**Figure 3.**
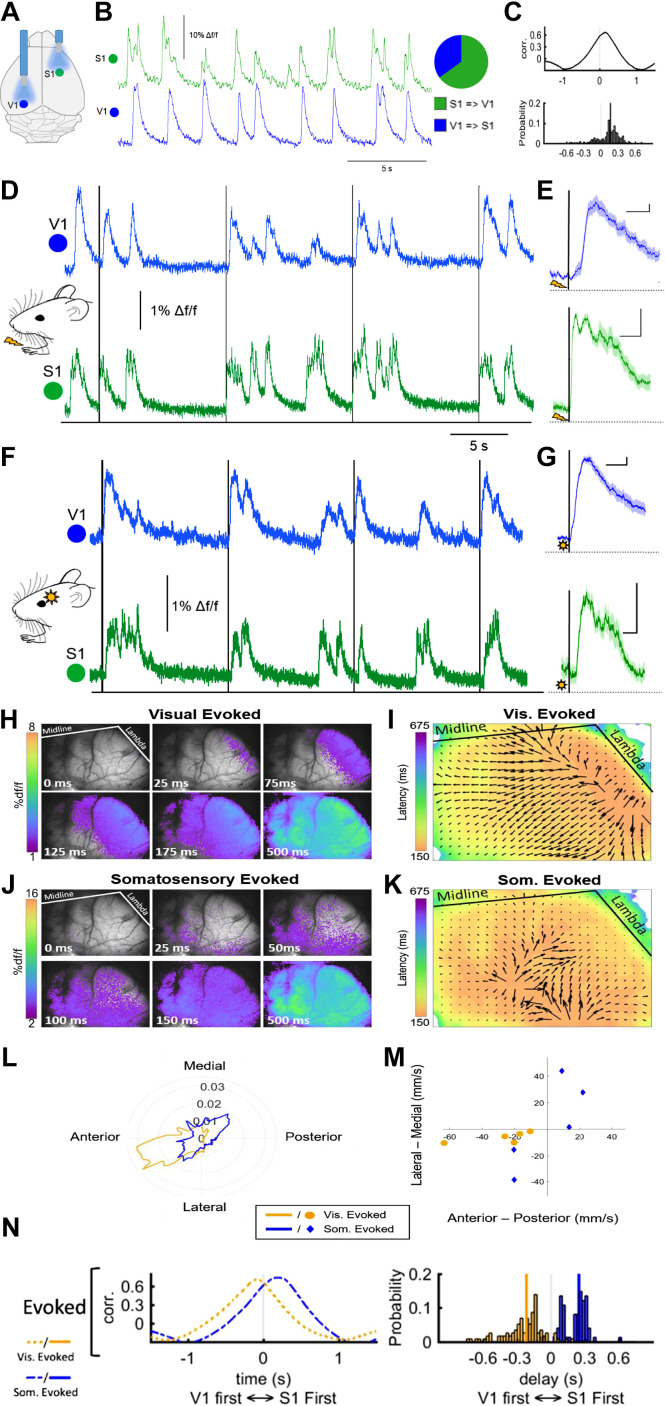
Sensory processing during slow wave activity (SWA) state. *A*: photometry-based calcium signals were measured in contralateral somatosensory (S1) and visual (V1) cortex as depicted (or in ipsilateral S1 and V1). *B*: ongoing slow wave events are synchronized between the 2 recording sites, with events detected in 60% (5 animals, 120 events) of the cases occurring first in S1, suggesting a propagation of waves in anterior-posterior direction. *C*: cross-correlogram of the 2 depicted signals indicated that the posterior waves follow the anterior ones with variable delays. *D*: stimulus-evoked slow wave events start in the area of their respective sensory afferents and are detected with stereotypic delays in distant recording sites. Forepaw stimulation [10-ms pulses of 1 mA, interstimulus interval (ISI) 10 s] reliably evokes slow wave events after 73 ms in S1 and after 290 ms in V1 (averages of 20 events each). *E*: averages of stimulus-locked traces shown in *D* (line = mean, shading = SE of 30 evoked events; vertical scale bars, 1% df/f; horizontal scale bars, 500 ms). *F*: visual stimulation (10-ms LED light flash, ISI 10 s) leads to waves occurring after a mean delay of 114 ms in V1 and 340 ms in S1 in the same animal (averages of 20 evoked events). *G*: averages of traces shown in *F* (line = mean, shading = SE of 30 evoked events; vertical scale bars, 1% df/f; horizontal scale bars, 500 ms). *H*: still frames from a df/f movie depicting the propagation of a visually evoked wave. *I*: quiver plot depicting pixel-by-pixel propagation velocities and direction for visual stimuli. *J*: still frames from a df/f movie depicting the propagation of a somatosensory-evoked wave. *K*: quiver plot depicting the pixel-by-pixel propagation velocities and direction for *J*. Color map indicates response latency for all pixels. *L*: weighted polar distribution of propagation velocities for visual and somatosensory stimuli for all animals. *M*: the vector mean propagation velocity and direction for each animal. *N*: correlation between V1 and S1 for evoked slow waves (*left*) and delay between V1 and S1 (*right*).

To further inform on the spatiotemporal properties of the described states and to probe the impact of functional state on cortical responses upon incoming information, population calcium photometry signals were recorded in S1 and V1 under the above-described sedated conditions (Fig. 3*A*). Here, simultaneous calcium recordings with optic fibers placed in S1 and contralateral V1 showed synchronous slow wave events in both regions (Fig. 3, *B* and *C*), with events being detected in S1 first in 60% of the cases. To further understand the directionality of slow wave propagation, we plotted the mean cross-correlation of S1 versus V1 for spontaneously occurring slow wave events (Fig. 3*C*), suggesting a propagation of waves in anterio-posterior direction as previously reported by others (29, 34, 35). Overall, spontaneous events had a mean delay of 128 ms from S1 to V1 [median: 167, interquartile range (IQR): 79.4–262, *n* = 217 events]. Propagation of waves was also observed for stimulus-induced slow wave events. Electric forepaw stimulation (1-mA pulses contralateral to the recording site; Fig. 3, *D* and *E*) evoked a slow calcium wave detected after a latency of 98 ± 7 ms (*n* = 141 events in 7 animals) in S1 and a mean latency of 204 ± 7 ms (*n* = 67 events in 3 animals) in ipsilateral V1 (in contralateral V1, waves were detected after a mean latency of 228 ± 8 ms; *n* = 81 events in 4 animals). Visually evoked slow waves (Fig. 3, *F* and *G*) were detected with a mean latency of 115 ms (±4 ms; *n* = 115 events in 7 animals) in V1 and a mean latency of 263 ms (±10 ms; *n* = 40 events in 3 animals) in ipsilateral S1 (in contralateral S1, waves were detected after a mean latency of 288 ± 11 ms; *n* = 71 events in 4 animals). The propagation velocity of these waves was on average 30 mm/s, similar to values reported previously (29). During PA, response latencies in the primary sensory area were much shorter, and stimulus-locked responses in the sensory area distant from the area of the primary sensory input (i.e., in S1 during visual stimulation or in V1 during forepaw stimulation) were not observed (Supplemental Fig. S3). When stimulus onsets were jittered, no meaningful signal changes with similar latencies could be identified for either of the conditions (Supplemental Fig. S4), speaking to the observed SWA and PA responses being stimulus locked. This is in line with previous work in rats that reported comparable latencies for both LFP and photometry responses upon forepaw stimulation, as well as their adaptation starting at stimulus frequencies above 5 Hz during PA (69). Given that fiber photometry only captures a small fraction of local cortical activity, we sought to better understand the relation between propagation patterns, sensory stimuli, and functional state with wide-field calcium imaging. Using transgenic animals expressing GCaMP, we obtained high-spatial resolution images of slow wave propagation throughout a majority of one hemisphere of the cerebral cortex at 20–40 Hz, employing thinned-skull imaging techniques (Fig. 3, *H* and *I*). Similarly to what was observed for the above-described dual-photometry measurements, waves appeared to initiate in the region corresponding to the sensory modality stimulated and then propagate across the cortex with stereotypic propagation direction and velocities (Fig. 3, *I–L*). To quantify the differences in propagation direction between stimuli, we compared the weighted distribution of velocity vectors (Fig. 3*L*). Visually and somatosensory-evoked responses differed significantly in their propagation direction distribution profile (*P* < 0.001), indicating different propagation patterns. The average propagation of visual evoked waves was consistently in the anterolateral direction, whereas somatosensory-evoked responses varied, with 3/5 propagating in the posteromedial direction and 2/5 propagating rostrolaterally (Fig. 3*M*). The velocities did not differ significantly when direction was disregarded (*P* = 0.78, Student’s *t* test) and had a combined mean of 30.5 ± 5.3 mm/s (*n* = 10). The specificity of initiation site and propagation direction but not velocity with respect to stimulus modality suggests that the SWA state is receptive to a range of incoming sensory inputs and favors the broad propagation of this signal across the cortex. To further understand the directionality of slow wave propagation, we plotted the mean cross-correlation of photometry signals from S1 and V1 for sensory-evoked stimuli (Fig. 3 *N*, *left*). The peak of the correlation has different sign depending on the stimulus, indicating that visual- versus somatosensory-evoked slow waves propagate in opposite directions. We also quantified the delay between event onset in V1 versus S1 for evoked events (Fig. 3 *N*, *right*). The delays for somatosensory stimuli appear to include two populations. The less prevalent, shorter delay time could represent slow waves evoked by another stimulus (i.e., an auditory noise in the room) that occurred nearly simultaneously with a somatosensory stimulus or other types of neural activation due to the intense electrical stimulus. Overall, the S1-to-V1 delay for somatosensory stimuli had a mean of 247 ms (median: 247.0, IQR: 105–292, *n* = 74) (*P* = 0.009), whereas the S1-to-V1 delay for visual stimuli was significantly slower with a mean of 445 ms (median: −218, IQR: −385 to −144; *P* < 0.001).

### Refractory Properties of SW Responses

**Figure 4.**
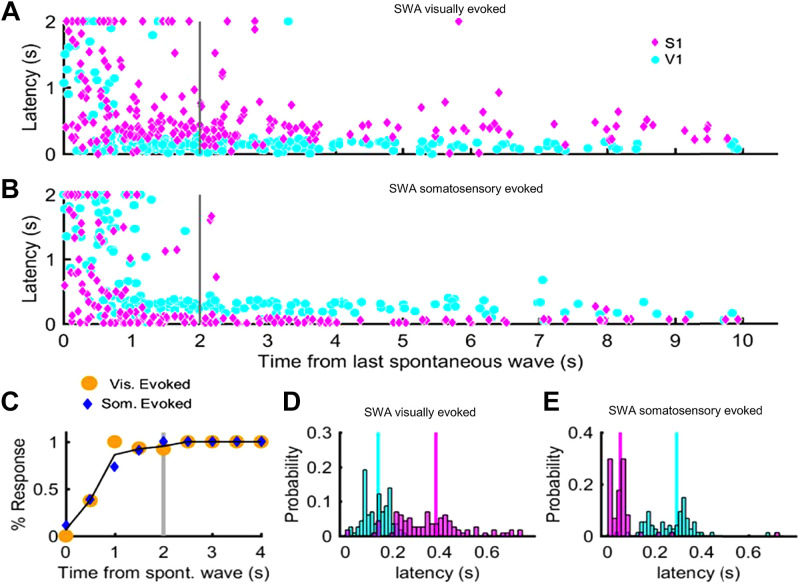
Effect of sensory stimuli at different intervals following spontaneous slow waves. *A* and *B*: latency to next detected slow wave and the time from last detected slow wave at time of visual and somatosensory stimulation, respectively. Variability in response time is reduced when stimuli are applied outside the refractory period (gray line), which is calculated in *C* as the time following a spontaneous slow wave after which 90% of stimuli are followed by a slow wave within 500 s. SWA, slow wave activity; S1, somatosensory cortex; V1, visual cortex. *D* and *E*: latency of calcium responses in S1 and V1 from time of visual and somatosensory stimulation, respectively. Only stimuli arriving after the refractory period are included.

Additionally, we observed an effect of sensory stimuli at different intervals following spontaneous slow waves (Fig. 4, *A* and *B*), indicating that slow wave events show refractory periods as previously shown (29). Somatosensory-evoked response latencies were faster than those of visual responses, and responses in the cortical area associated with the stimulus were detected significantly sooner for somatosensory versus visual stimuli (*P* < 0.001). Overall response time variability is reduced when stimuli are applied outside the refractory period, calculated as the time following a spontaneous slow wave after which 90% of stimuli are followed by a slow wave within the next 500 s (Fig. 4*C*). Yet the waves that occur after the refractory period show different response latencies in S1 and V1 from time of visual and somatosensory stimulation, respectively (Fig. 4, *D* and *E*).

### Primary Responses in the Corresponding Sensory Area Precede a Propagating Global Wave

**Figure 5.**
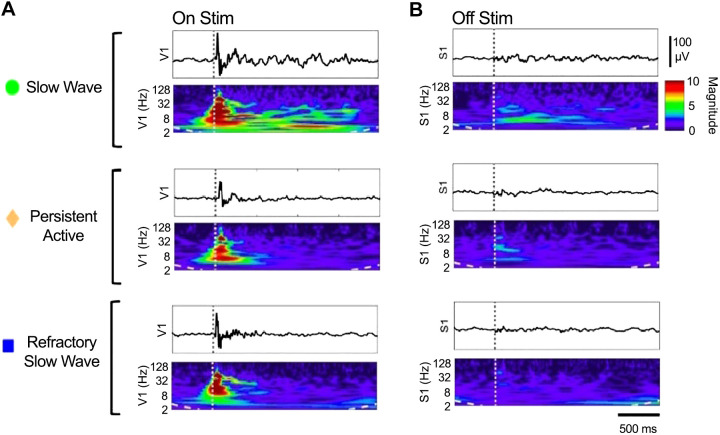
Primary responses in the stimulus-encoding sensory area occur before the onset of an evoked slow wave activity (SWA) event. *A*: electrical recordings of slow waves in 1 animal under 3 conditions: stimulus-triggered averages in visual cortex (V1) (On Stim). *B*: simultaneous recordings in somatosensory cortex (S1) (Off Stim). Corresponding spectrograms of responses displayed beneath each trace. Dotted line marks the onset time of the visual stimulus.

We showed that slow wave activity is dominated by stereotypically reoccurring slow wave events. Although reliably elicited by peripheral stimulation, the microarchitecture of slow wave events and their stereotypical shape does not seem to encode stimulus features, which might be related to the engagement of large areas of the cortex during this state. As spectral analysis of electrical recordings is able to separate sensory response components, we used intracranial electrode implants to record local field potentials in V1 and S1 in response to visual stimuli. We compared the spectral content of responses to visual stimulation when the cortex was in SWA and PA states. In addition, we also examined a subset of responses that occurred during the refractory window observed in SWA state. Example stimulus response averages and quantification in log(fold change) are shown in Fig. 5, with summary values and statistics of the electrical response shown in Table 1 and Table 2. During SWA state we observed an initial high burst activity with broad spectral content delta through gamma, which was time locked to the stimulus (Fig. 5*A*). This initial burst was then followed by a 2- to 3-s period of sustained elevation in delta through beta but not gamma activity. Upon examining the cortical area being stimulated (V1) we found that this primary fast response was present in the electrophysiology data of both SWA and PA states as well as during the refractory window of SWA state (Fig. 5*A*). This was quantified by comparing the gamma power responses between these states in V1, which were all elevated in response to stimulation but did not differ significantly from each other (Fig. 5*A*; Table 1, Table 2). We then examined the response to visual stimuli in somatosensory cortex (Fig. 5*B*). In S1 we found that only SWA state allowed propagation of visual information across the cortex. In SWA state S1 exhibited a sustained lower-frequency response similar to a secondary response in V1 but not the higher-frequency gamma response. Neither the primary nor the secondary responses were observed in S1 during PA state or in the refractory window of SWA state (Fig. 5; Table 1, Table 2). Collectively, these findings suggest two independent phenomena that are differentially regulated by cortical state and are carried by cortical oscillations distinct in frequency and time course.

**Table 1.** Visual response properties

Frequency	Region	Brain State	Mean, log(FC)	Median, log(FC)	IQR, log(FC)	*n* (animals)
Total power (0.5–200 Hz)	V1	SW	2.68	2.42	2.16–3.34	10
		SW-r	0.67	0.39	−2.6e−3 to 1.20	6
		PA	1.83	1.94	1.40–2.46	10
	S1	SW	2.00	1.78	1.26–2.78	10
		SW-r	−0.36	−0.43	−0.71 to −0.19	6
		PA	0.61	0.52	0.38–1.02	10
Sub-gamma (0.5–35 Hz)	V1	SW	2.69	2.69	2.12–3.12	10
		SW-r	0.56	0.31	−0.12 to −1.09	6
		PA	1.79	1.84	1.46–2.25	10
	S1	SW	2.06	1.80	1.24–3.03	10
		SW-r	−0.37	−0.54	−0.91 to −0.13	6
		PA	0.61	0.56	0.42–0.96	10
Gamma (35–200 Hz)	V1	SW	2.20	2.26	1.19–2.90	10
		SW-r	1.53	1.58	0.76–2.02	6
		PA	2.08	2.17	1.20–2.92	10
	S1	SW	1.16	0.80	0.52–1.66	10
		SW-r	−0.19	−0.29	−0.43 to 0.15	6
		PA	0.55	0.62	0.18–0.78	10

FC, fold change; IQR, interquartile range; PA, persistent activity; SW, slow wave; SW-r, refractory slow wave; S1, somatosensory cortex; V1, visual cortex.

**Table 2.** Visual response significance tests

Comparison	Sub-Gamma (0.5–35 Hz)	Gamma (35–200 Hz)	Total
V1 vs. S1 Wilcoxon signed rank			
SW	*P* = 0.055	*P = 0.020	*P = 0.020
SW-r	*P = 0.031	*P = 0.031	*P = 0.031
PA	**P = 0.004	**P = 0.002	**P = 0.004
V1 Mann–Whitney *U*			
SW vs. PA	**P = 0.008	*P* = 0.968	**P = 0.053
SW-r vs. SW	**P = 0.002	*P* = 0.456	**P = 0.002
SW-r vs. PA	**P = 0.011	*P* = 0.368	**P = 0.016
S1 Mann–Whitney *U*			
SW vs. PA	**P < 0.001	*P* = 0.243	**P < 0.001
SW-r vs. SW	** *P* = 0.012	*P* = 0.328	**P = 0.026
SW-r vs PA	** *P* = 0.011	** *P* = 0.005	**P = 0.011

PA, persistent activity; SW, slow wave; SW-r, refractory slow wave; S1, somatosensory cortex; V1, visual cortex.

## DISCUSSION

Distinct functional states engage different areas of the brain and depending on their spatial extension shape representations of sensory afferents (9, 10). Considering that state transitions can occur under a variety of conditions (70), it is of importance to first characterize functional state as a variable by investigating the related ongoing neuronal signals across different cortical scales. Second, it is of interest to test how functional states shape the afferent output of the circuits they dominate. Functional states generated through a modulation of internal activity, independent of environmental stimuli, may bring along different excitability modes of neuronal networks and are thereby likely linked to distinct processing of information. Hence, under anesthesia as well as in awake conditions, the current functional state of the cortical network provides a rich experimental variable able to explain distinct properties of neuronal responses (6). Anesthesia-related states are of relevance for neurophysiological investigations, both as pharmacological proxies for investigating the features and dynamics of unconscious states (42) as well as for rodent neuroimaging studies. For the latter, especially for functional magnetic resonance imaging studies, animals usually have to be anesthetized or sedated to prevent motion artifacts that can affect neural dynamics (55, 56) and potentially distort imaging data. It has previously been shown that different anesthesia regimens lead to different whole brain signatures. Different types of anesthesia can influence whole brain responses (71, 72) or local hemodynamic responses (73) to sensory stimuli and can lead to different resting-state functional connectivity networks (74, 75). Studies taking into account the related underlying state of cortical or thalamic neuronal populations demonstrated that these locally recorded signals themselves can predict whole brain activity (20) and functional connectivity (15). It has further been shown that local recordings of neuronal activity carry information that allows decoding of cortex-wide brain activity across modalities (76). We reported here that the two exemplary states, SWA and PA, differ regarding spatiotemporal characteristics on the local and global scales and show state-dependent propagation modes, which might explain these findings. The two states represent an experimental model for cortical information processing during conditions with similar features and neuronal signature that can occur either under other types of anesthesia or during behavioral changes in the awake brain. The underlying state of a neural network is relevant for explaining response variability of neural networks under seemingly constant conditions, as physiological state can vary largely over experiments with long duration or during certain types of anesthesia as for example urethane (9, 42). A state dominated by slow waves similar to our defined SWA state can occur under different types of anesthesia, for example isoflurane, pentobarbital, and propofol (42) or ketamine-xylazine (68). In this state of unconsciousness and cortical disconnection, synchronous slow waves likely originate from layer 5 pyramidal neurons (28, 68), which switch to a mode characterized by highly active, but spatially synchronous outputs, with seemingly low information content (68). It is therefore likely that the sensory triggered waves carry no information regarding their sensory content but merely originate and then propagate from the sensory area that was stimulated (23), not unlike waves originating from a stone being thrown in a quiet lake (33, 77). Our findings presented here indicate that the response of the cortical network to a sensory stimulus critically depends on the current functional state of the network. Whereas neuronal activity upon sensory stimulation stays local during PA state, during SWA state a traveling slow wavefront, with its initiation and direction of propagation depending on the sensory modality stimulated, can be observed over large areas of the cortex. Sensory responses during SWA versus PA state are more variable, with propagating waves often, in an all-or-none fashion, triggered by stimuli during SWA state but not during a refractory period following individual slow waves. Spectral analysis of LFP recordings showed that gamma activity (>40 Hz) is always observed in the cortical area associated with the stimulus.

### Mesoscale Correlates of Functional States

Population activity measured via optical fiber recordings has allowed previous investigations of slow wave events in anesthetized animals (29, 53, 78). However, lightly anesthetized and awake recordings reveal a persistently active state in which cortical activity occurs continuously, without the periods of quiescence that are characteristic of SWA state. Upon peripheral somatosensory stimulation during SWA, stereotypical calcium and LFP deflections were observed, which were time-locked to the stimulation pulses but similar to spontaneously occurring events regarding their properties. During PA state, the same stimulus pulses led to short-latency responses that precisely followed upon each single pulse. As changes of functional state can occur spontaneously in awake state as well as during prolonged anesthesia (42), they may appear as noise if they are disregarded in the statistical modeling of neural signals (6). When considered, changes of functional state can contribute to explaining variability in experimental results under a variety of conditions.

### Sensory Responses in PA and SWA State

Our results indicate that the direction of slow wave propagation depends on the sensory stimuli used to trigger such waves. Given that visually evoked waves initiate posteriorly and propagate in anterior direction whereas somatosensory stimuli initiate more anterior and to posterior sites, it appears that stimulation of different sensory modalities activates waves in associated cortices that are able to propagate depending on the refractoriness of other regions. The generally observed anterior-posterior propagation of slow waves (32, 34) may reflect that the dominant sensory pathways activated in wakefulness, and thus subsequently during slow-wave sleep, are located in more anterior regions.

### Refractory Period

EEG studies have indicated that slow waves may be activated locally and can propagate broadly depending on whether the brain is stimulated in phase with the natural slow oscillation (32, 34). The present study indicates that the phase of the network activity reflects a propensity of the slow wave-generating network toward refractoriness following active periods of the SW state. The dependence of SW generation on network history is likely to explain some of the wide variability of sensory responses observed when calcium imaging techniques are used to measure cortical activity in vivo. Not only does the cortex display distinct states with different sensory response modes, the cortical response to stimuli can vary greatly within one state, depending on the sensory modality stimulated and the history of previous activity in the network. Still, functional state may gate the secondary, but not the primary, response to sensory stimulation. We observed a fast, high-frequency primary response encoded in the stimulated sensory area, independent of cortical state, followed by a secondary, slow and sustained low-frequency response that occurs and propagates only during SWA state. These primary responses likely reflect incoming primary sensory information, presumably through thalamo-cortical projections. It is also interesting to note that the putative cortico-cortical activity appears to propagate via rhythms such as delta and theta that are associated with cortical synchrony in states as SWS (79, 80).

In conclusion, we put forward the notion that the spatiotemporal characteristics of both spontaneous and stimulus-evoked responses in the cortex are governed by rather distinct functional states, putatively serving two distinct neurophysiological roles. SWA state here likely indicates cortex-wide synchronicity as a signature of anesthesia-related unconsciousness, at least in rodents (33), lacking processing of incoming sensory information (68), but a similarly synchronous state might be required for memory consolidation or synaptic homeostasis (81) during SWS (77). PA state allows for selective, modality-specific activation, which is required for a nuanced percept. When comparing common anesthesia regimens in rodents in terms of their ongoing activity on the mesoscale, it is not always apparent which physiological (e.g., sleep) or pathological (e.g., coma) condition they might represent (42). As demonstrated by the joint occurrence of primary and secondary responses in SWA state, aspects of these brain states can be identified in various conditions. SWA and PA are likely not exclusive to a specific anesthetic, behavioral, or physiological condition. Including a state variable for neurophysiological and neuroimaging data might therefore result in less variance in a given data set. A brain state-informed analysis might lead to a better understanding of the respective valence of a neural activation pattern, which is of particular relevance in translational studies.

## SUPPLEMENTAL DATA

Supplemental Videos S1 and S2 and Supplemental Figures S1–S4: https://github.com/Strohlab/Schwalm_et_al_JNP_2022.

## GRANTS

This research was supported by a Royalty Research Fund of the University of Washington. D.R.T. was supported by a Neuroscience Training Grant (T32NS099578) and by the Computational Neuroscience Training Grant. C.E. was funded by a DAAD postdoctoral exchange scholarship. A.S. was supported by the Boehringer Ingelheim Foundation and the German Research Foundation (DFG).

## DISCLOSURES

No conflicts of interest, financial or otherwise, are declared by the authors.

## AUTHOR CONTRIBUTIONS

M.S., P.M., W.J.M., and A.S. conceived and designed research; M.S., D.R.T., C.E., T.J.R., and H.W. performed experiments; M.S., D.R.T., and C.E. analyzed data; M.S., D.R.T., and C.E. prepared figures; M.S., D.R.T., C.E., and A.S. drafted manuscript; W.J.M. and A.S. edited and revised manuscript; M.S., D.R.T., C.E., T.J.R., P.M., H.W., W.J.M., and A.S. approved final version of manuscript.
